# Amyloid β aggregation induces human brain microvascular endothelial cell death with abnormal actin organization

**DOI:** 10.1016/j.bbrep.2021.101189

**Published:** 2021-12-15

**Authors:** Yushiro Take, Yusaku Chikai, Keiya Shimamori, Masahiro Kuragano, Hiroki Kurita, Kiyotaka Tokuraku

**Affiliations:** a Graduate School of Engineering, Muroran Institute of Technology, Hokkaido, 050-8585, Japan; b Ohkawara Neurosurgical Hospital, Hokkaido, 050-0082, Japan; c Department of Cerebrovascular Surgery, International Medical Center, Saitama Medical University, Saitama, 350-1298, Japan

**Keywords:** Amyloid β, Blood-brain barrier, Cerebral amyloid angiopathy, Endothelial cell, Quantum dots, Aβ, amyloid β, CAA, cerebral amyloid angiopathy, hBMEC, human primary brain microvascular endothelial cell, QD, quantum dot

## Abstract

Cerebral amyloid angiopathy (CAA) is a disease in which amyloid β (Aβ) is deposited on the walls of blood vessels in the brain, making those walls brittle and causing cerebral hemorrhage. However, the mechanism underlying its onset is not well understood. The aggregation and accumulation of Aβ cause the occlusion and fragility of blood vessels due to endothelial cell damage, breakdown of the blood-brain barrier, and replacement with elements constituting the blood vessel wall. In this study, we observed the effect of Aβ on human primary brain microvascular endothelial cells (hBMECs) in real-time using quantum dot nanoprobes to elucidate the mechanism of vascular weakening by Aβ. It was observed that Aβ began to aggregate around hBMECs after the start of incubation and that the cells were covered with aggregates. Aβ aggregates firmly anchored the cells on the plate surface, and eventually suppressed cell motility and caused cell death. Furthermore, Aβ aggregation induced the organization of abnormal actin, resulting in a significant increase in intracellular actin dots over 10 μm^2^. These results suggest that the mechanism by which Aβ forms a fragile vessel wall is as follows: Aβ aggregation around vascular endothelial cells anchors them to the substrate, induces abnormal actin organization, and leads to cell death.

## Highlights

• Amyloid β (Aβ) aggregates anchor human endothelial cells to the substrate.
• Aβ induces abnormal actin organization in human endothelial cells.
• Aβ induces cell death of human endothelial cells.

## Graphical abstract

**Figure:**
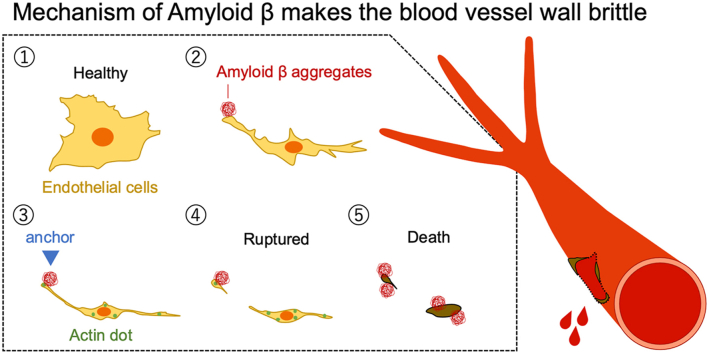

## 1 Introduction

The aggregation and accumulation of amyloid β (Aβ) in the brain triggers the development of Alzheimer's disease, but its aggregation in cerebral microvessels is also involved in the development of cerebral amyloid angiopathy (CAA). CAA is a major cause of spontaneous intracerebral hemorrhage in elderly people and plays an important role in cognitive decline in this sector of the population [1]. The deposition of amyloid around perivascular, small arteries and capillaries of the leptomeninges and cerebral cortex causes cerebral hemorrhage [2,3]. During perivascular drainage of interstitial fluid, pulsation of blood vessels plays an important role in the excretion of Aβ [4,5]. In a mouse model, impaired vascular pulsation markedly reduced Aβ clearance and increased local Aβ deposition [6,7]. It has been suggested that the deposition of amyloid on the vascular wall may cause the dysfunction of vascular endothelial cells and the blood-brain barrier [8,9]. As mentioned above, although Aβ aggregation and accumulation in microvessels induces brain dysfunction, for various reasons, the mechanism of vascular fragility and endothelial cell damage due to Aβ deposition is not clear.

We previously reported a real-time imaging method of Aβ aggregation using quantum dot (QD) nanoprobes [10]. In this imaging method, Aβ aggregates can be visualized by fluorescence microscopy by adding 0.1–0.01% of QD-labeled Aβ to unlabeled Aβ [10]. Subsequently, we developed a microliter scale high-throughput screening system for Aβ aggregation inhibitors that applies this imaging method [11], and screened various aggregation inhibitors [[12], [13], [14], [15], [16]]. This QD-based imaging method has also been applied to visualize the aggregation process of tau, α-synuclein, and serum amyloid A [17,18]. Since QD can be imaged for a long period of time due to its high photostability, we also attempted to image the process of Aβ aggregation in the presence of cultured cells using a QD nanoprobe. We succeeded in the real-time imaging of the entire process from Aβ aggregation around PC12 cells to the induction of cell death by apoptosis [19].

In this study, we attempted to analyze, in real-time, the relationship between the Aβ aggregation process and changes in vascular endothelial cell morphology using QD nanoprobes to investigate the effects of Aβ aggregation and accumulation on endothelial cells. Furthermore, we successfully visualized how Aβ aggregates damaged human primary brain microvascular endothelial cells (hBMECs).

## 2 Materials and methods

### 2.1 Materials

Human Aβ_42_ (4349-v; Peptide Institute) and Cys-conjugated Aβ_40_ (23519; Anaspec) were purchased commercially. QD-PEG-NH_2_ (Qdot 655 ITK Amino (PEG) Quantum dot; Q21521MP) and Alexa 488-Phalloidin (A12379) were purchased from Thermo Fisher Scientific. QDAβ was prepared according to our previous reports [10,19]. hBMECs and human endothelial cell medium (no phenol red) kit were purchased from Cell Biologics.

### 2.2 Imaging of Aβ aggregation with cultured endothelial cells

hBMECs were maintained in human endothelial cell medium (no phenol red) supplemented with 0.1% VEGF, 0.1% heparin, 0.1% EGF, 0.1% hydrocortisone, 0.1% FGF, 1% l-glutamine, 1% antibiotic-antimycotic solution and 5% fetal bovine serum. Cells were cultured at 37 °C in humidified air containing 5% CO_2_. Pre-cultured cells were plated at 1000 or 5000 cells in a glass-bottomed 96-well plate (5866–096, IWAKI) precoated with 0.3 mg/cm^2^ gelatin (01393-100 ML, Sigma-Aldrich). After incubation for 24 h, medium was removed from each well and wells were refilled with new medium including DMSO (control) or Aβ_42_ in DMSO and QDAβ. Time-lapse images were captured with an inverted microscope (Ti-E; Nikon equipped with a color CMOS camera (DS-Ri2; Nikon) and an objective lens (PlanApo λ 20 × /0.75 NA; Nikon) and standard TRITC (TRITC-A-Basic-NTE, ex: 532–552 nm, em: 594–646 nm) filter sets (Semrock). During observation, the cells were warmed in a chamber set at 37 °C (INUBTF-WSKM-B13I; Tokai Hit). Images were captured every 10 min and analyzed using NIS-Elements AR software (Nikon). The resulting data was edited by ImageJ software (NIH).

### 2.3 Fluorescence microscopic observations

hBMECs incubated with Aβ_42_ and QDAβ were fixed in 3.7% formaldehyde for 20 min. After washing with PBS, cells were stained by 0.66 μM Alexa 488-phalloidin for 1 h. Fluorescence microscopic images were captured with an inverted microscope (Ti-E; Nikon) equipped with a color CMOS camera (DS-Ri2; Nikon) and standard TRITC (TRITC-A-Basic-NTE, ex: 532–552 nm, em: 594–646 nm) and FITC (FITC-A-Basic-NTE, ex: 457–492 nm, em: 508–551 nm) filter sets (Semrock). To quantify abnormal actin dots in hBMECs, images were analyzed using ImageJ software (NIH). First, the actin dots observed in hBMECs were surrounded by the ImageJ “Freehand” function, and the area and number of all dots were measured. Next, the actin dots in the range of 5–100 μm^2^ were selected to remove small spots that could not be judged as actin as well as other contaminants in the medium. Thus, in this paper, we defined actin dots in the range of 5–100 μm^2^ as abnormal actin dots. Cell area was measured using the “Analyze Particles” function in ImageJ after conversion to an 8-bit grayscale and adjustment using the “Threshold” function. We used these data to quantify the number of abnormal actin dots per cell area (dots/μm^2^). 3D images of Aβ aggregates and cells were captured using a confocal laser microscope system (C2 Plus; Nikon) equipped with an objective lens (Plan Apo λ 4 × /0.20 NA; Nikon, Plan Apo λ 20 × /0.75 NA; Nikon). Apoptosis was detected using the pSIVA™ Real-Time Apoptosis Fluorescent Microscopy Kit (APO004; Bio-Rad) according to manufacturer's procedure. The fluorescence intensity of pSIVA observed using an inverted microscope (Ti-E) was measured from each fluorescence image using the “mean gray value” of ImageJ software. The obtained data groups were tested for significance by a *t*-test using Excel (Version 16; Microsoft).

## 3 Results

### 3.1 Real-time imaging of cell death induced by Aβ aggregation

**Fig. 1:**
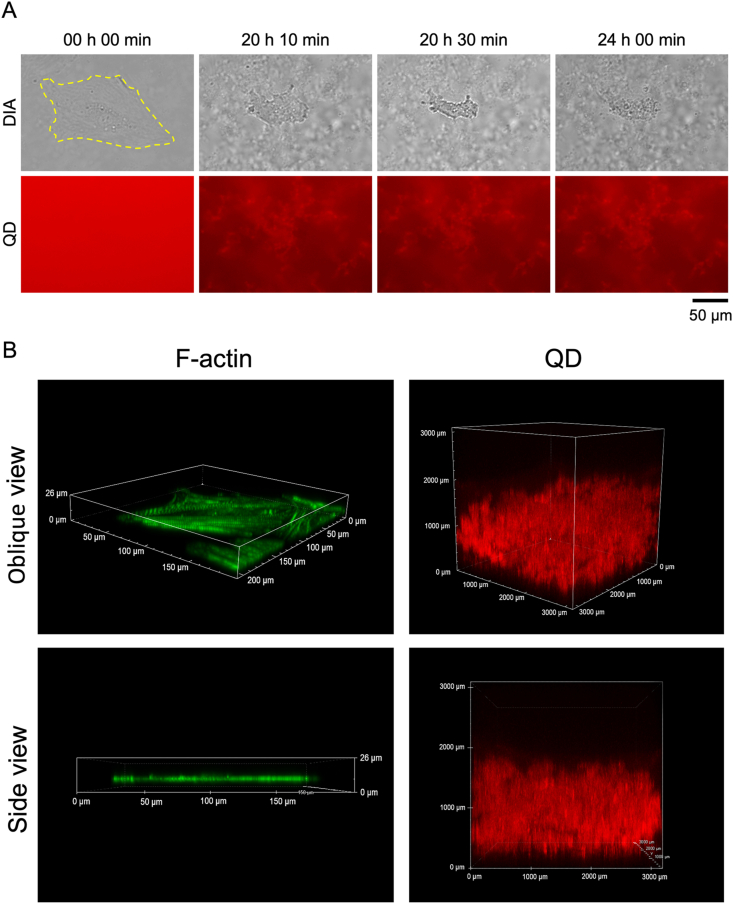
Real-time imaging of Aβ aggregation and endothelial cell death. (A) hBMECs co-incubated with 20 μM Aβ_42_ and 30 nM QDAβ were observed by an inverted fluorescence microscope. Time series of images shows the gradual steps of Aβ_42_ aggregation and cell death. The yellow dotted line indicates the outline of an endothelial cell. (B) Left panel; 3D images of an hBMEC stained with F-actin with Alexa488 phalloidin. Right panel; 3D images of Aβ aggregates co-incubated under the same conditions as in (A). Images were captured by a confocal microscope. (For interpretation of the references to color in this figure legend, the reader is referred to the Web version of this article.)

First, we observed hBMECs co-incubated with 20 μM Aβ_42_ and 30 nM QDAβ using an inverted fluorescence microscope. The added QDAβ concentration only accounted for 0.1% of Aβ. We previously confirmed that the effect on cultured cells under these conditions mainly reflects the effect of added Aβ [15]. Real-time imaging showed that Aβ aggregates began to be observed about 1 h after the start of incubation and that aggregation became saturated in about 10 h (Sup. Movie. S1). Aβ aggregates were observed around hBMECs (Fig. 1), similar to our recent report using PC12 cells [19]. A time series of images demonstrates the gradual steps of Aβ aggregation and cell death with cell shrinkage after 20 h of incubation (Fig. 1). Cell death with shrinkage due to Aβ aggregation was observed in other areas after 10–20 h of incubation (Sup. Fig. S1). Cells were actively moving after incubation for 24 h in the control condition without Aβ (Sup. Movie S2), but almost no cell movement was observed after 10 h of incubation when whole cells were covered with Aβ (Sup. Movie S1). Cells were several μm thick while that thickness of Aβ aggregates was about 1000 μm, suggesting that most of the Aβ aggregates observed in Fig. 1A had aggregated in the extracellular space. This result suggests that Aβ aggregation around the cell suppresses cell motility.

Supplementary data related to this article can be found at https://doi.org/10.1016/j.bbrep.2021.101189.

The following are the supplementary data related to this article:

Multimedia component 2 Multimedia component 2

Multimedia component 3 Multimedia component 3

### 3.2 Anchorage of cells on plate surface and reduction of cell plasticity by Aβ aggregates

**Fig. 2:**
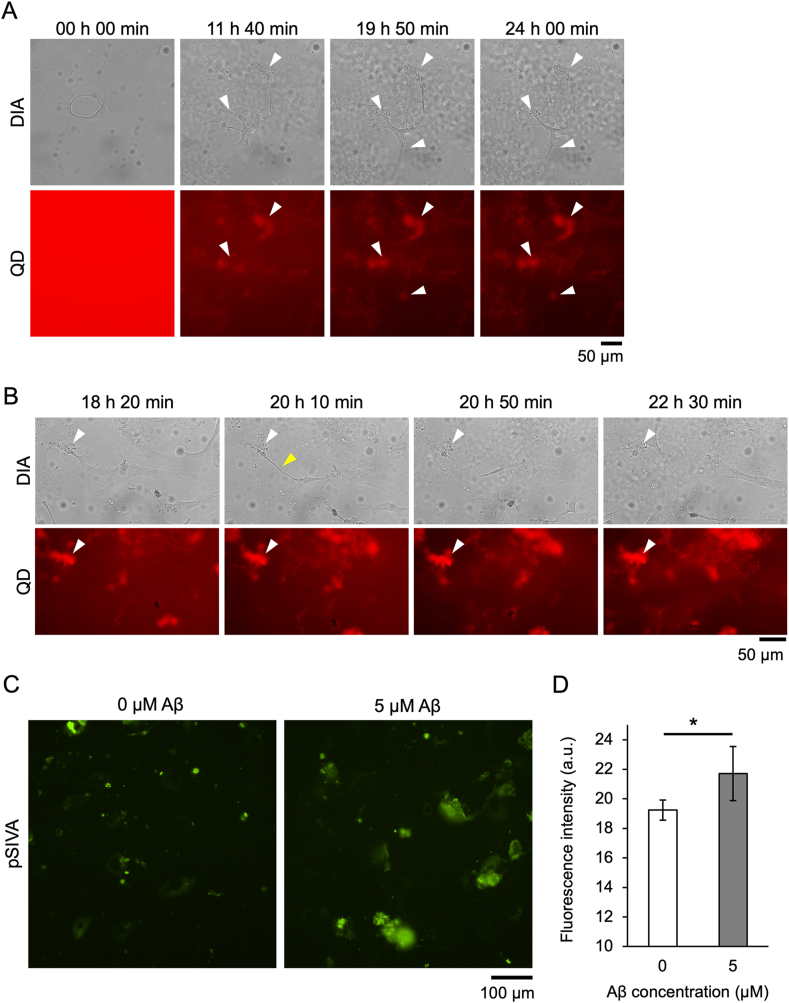
Anchoring of endothelial cells on a plate surface by Aβ aggregates. hBMECs co-incubated with 10 μM (A) or 5 μM (B) Aβ_42_ and 30 nM QDAβ were observed by an inverted fluorescence microscope. White arrowheads indicate points of cells anchored to the plate surface by Aβ_42_ aggregates. Yellow arrowhead indicates the point where the cell broke off. (C) Representative images of fluorescence observations for the pSIVA apoptosis marker in hBMECs co-incubated (24 h) with 0 and 5 μM Aβ. (D) The fluorescence intensity of pSIVA was estimated from each image (C) using Image J software. Since the average fluorescence intensity at 0 h incubation under each condition was around 10, the minimum value on the vertical axis was set to 10. The data represent the mean ± SD from five independent fields of view. * Shows a statistically significant difference by a two-sided *t*-test with 0.01 < *p* < 0.05. (For interpretation of the references to color in this figure legend, the reader is referred to the Web version of this article.)

When hBMECs were co-incubated with 10 μM Aβ_42_ (half the concentration in Fig. 1), cells were anchored to the plate surface at the Aβ aggregate-bound region (Fig. 2A and Sup. Movie S3, white arrowheads). The anchored region never became dissociated from the surface, suggesting that the adhesion of cells to the substrate by Aβ aggregates was so strong that they could not be peeled off by cell motility. When hBMECs were co-incubated with 5 μM Aβ_42_ (25% concentration in Fig. 1), cells were anchored to the plate surface at only one region to which Aβ aggregates bound (Fig. 2B and Sup. Movie S4, white arrowhead). The partially anchored cells continued to move and were finally ruptured by the movement of the cells themselves (Fig. 2B, yellow arrowhead). After cleavage, endothelial cells died. Cell death was evaluated using the apoptosis marker pSIVA (Fig. 2C), which confirmed that cell death was significantly induced in the presence of Aβ_42_ (Fig. 2D).

Supplementary data related to this article can be found at https://doi.org/10.1016/j.bbrep.2021.101189.

The following are the supplementary data related to this article:

Multimedia component 4 Multimedia component 4

Multimedia component 5 Multimedia component 5

### 3.3 Destruction and perforation of endothelial cell layer by Aβ aggregation

**Fig. 3:**
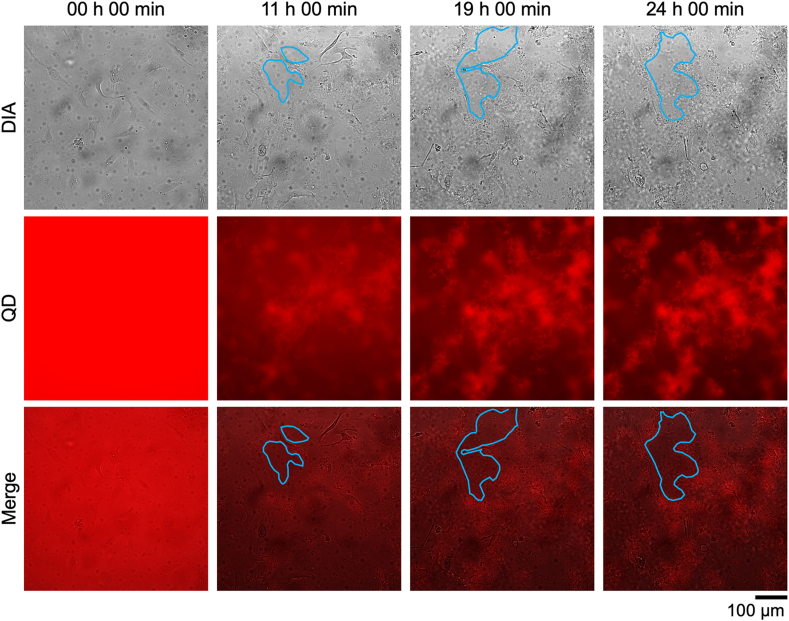
Holes in a cell monolayer induced by Aβ aggregation. hBMECs co-incubated with 10 μM Aβ_42_ and 30 nM QDAβ were observed by an inverted microscope. The areas surrounded by blue lines indicate the perforated areas in the cell monolayer. (For interpretation of the references to color in this figure legend, the reader is referred to the Web version of this article.)

When 10 μM Aβ was added to hBMECs cultured in confluence, the aggregate destroyed the cell layer, although it is unclear if this is a monolayer with a tight junction, and punctured it (Fig. 3, Sup. Movie S5). There were no holes in the cell layer at the beginning of incubation, and the holes enlarged as Aβ aggregation progressed. Hole size changed little after about 20 h when Aβ aggregation was saturated and cell motility stopped.

Supplementary data related to this article can be found at https://doi.org/10.1016/j.bbrep.2021.101189.

The following is the supplementary data related to this article:

Multimedia component 6 Multimedia component 6

### 3.4 Abnormal actin organization induced by Aβ aggregation

**Fig. 4:**
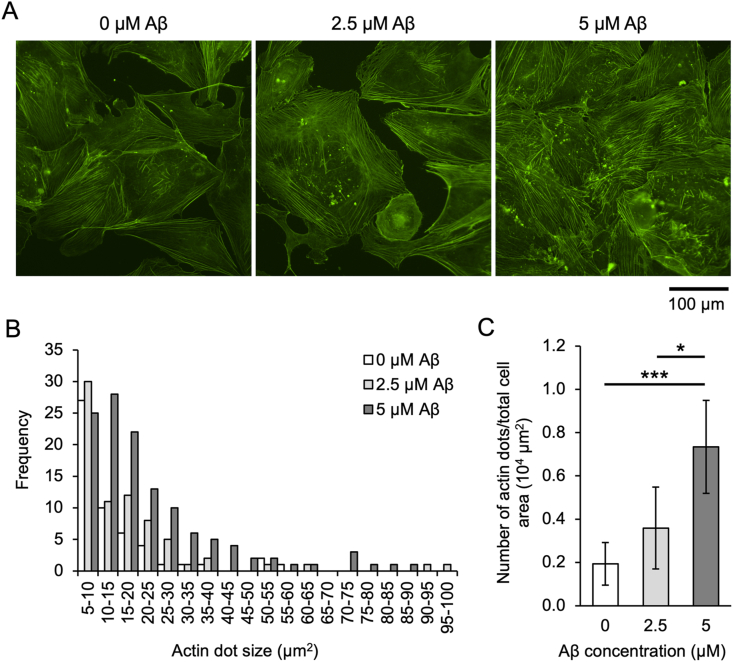
Aβ induces abnormal actin dots in endothelial cells. (A) Fluorescent images of hBMECs stained for F-actin (green) with Alexa 488 Phalloidin. hBMECs were co-incubated with Aβ of each concentration and 30 nM QDAβ for 24 h, stained with phalloidin, then imaged by an inverted microscope. (B) Histogram of abnormal actin dots estimated from the fluorescent images. Fluorescent images were analyzed using ImageJ software. The abnormal actin dots of 5–100 μm^2^ were counted for every 5 μm^2^. Data represent the total number from five independent visual fields. (C) The number of abnormal actin dots of 10 μm^2^ or more was normalized by cell area. Data represent mean ± SD from five independent visual fields. * and *** indicate statistically significant differences by a two-tailed *t*-test at 0.01 < *p* < 0.05 and *p* < 0.001, respectively. (For interpretation of the references to color in this figure legend, the reader is referred to the Web version of this article.)

Since it was revealed that Aβ aggregation affects the motility of hBMECs (Fig. 1, Fig. 2, Fig. 3), we next observed changes in the actin cytoskeleton involved in cell motility (Fig. 4). The results show that the abnormal organization of the actin cytoskeleton (actin dots) increased proportionally with the concentration of Aβ added (Fig. 4A). Quantification of dot size and number revealed that small dots of 5–10 μm^2^ occurred at the same frequency, regardless of Aβ concentration, although the frequency of 10–50 μm^2^ dots increased with increasing Aβ concentration (Fig. 4B). The number of dots in cells larger than 10 μm^2^ increased significantly, depending on the Aβ concentration (Fig. 4C). Some Aβ aggregates and actin dots were not completely co-localized, suggesting that actin dots are formed by the indirect effect of Aβ aggregation (Sup. Fig. S2).

## 4 Discussion

In this study, we found that Aβ aggregates strongly anchor hBMECs and substrates, like an adhesive (Fig. 1, Fig. 2, Fig. 3). The endothelial cells could not escape the Aβ adhesiveness using their own cell movement and were immobilized until cell death was induced (Fig. 1). Interestingly, it was also found that the adhesion of cells to the substrate by Aβ aggregates was greater than the force by which the cells were ruptured, and that partially anchored endothelial cells on the plate surface were cleaved by their own cell movement (Fig. 2B).

As Aβ aggregation progressed, the movement of vascular endothelial cells was suppressed, and finally, cell death was induced (Fig. 1). In this process, organization of the actin cytoskeleton became abnormal, and actin dots were observed (Fig. 4). Some of the actin dots appeared to be co-localized with Aβ aggregates, but many non-co-localized dots were also observed (Sup. Fig. S2). What is the mechanism by which abnormal actin cytoskeleton organization is indirectly induced by Aβ? In recent years, focus has been placed on the function of actin filaments as a mechanosensory device [20]. For example, it has been reported that myosin II shows a high affinity for tensioned actin filaments [20] and cofilin does not easily bind to it [21]. These actin-binding proteins play an important role in organizing the actin cytoskeleton. Tension in intracellular actin filaments by anchoring with Aβ aggregates affects the interaction between actin-binding proteins and the actin cytoskeleton, and this may lead to abnormal organization of the actin cytoskeleton. How Aβ aggregation affects the actin cytoskeleton is a topic for future exploration.

This study revealed that Aβ aggregation induces endothelial cell death with abnormal actin organization, ultimately disrupting the cell monolayer. Although actual Aβ concentration in blood is lower than the experimental conditions used in this study [[22], [23], [24]], Aβ deposition in cerebral microvessels has been observed [8]. In CAA, the accumulation of Aβ leads to cerebral endothelial cell dysfunction and death [25]. Additionally, not only does the accumulation of Aβ induce alterations of smooth muscle and endothelial cell layers, so too do amyloid deposition and concomitant microhemorrhages also occur in small capillary vessels lacking a smooth muscle layer. Aβ_42_ is the first species to be deposited in the vessel wall while vascular deposits also contain Aβ_42_ [26,27]. Moreover, the ratio of Aβ_40_:Aβ_42_ in capillary deposits is lower than in arteries and veins [[27], [28]]. These locally deposited Aβ aggregates interact with the surrounding vascular endothelial cells, presumably causing the destruction of the cellular layer, as was observed in this study. Therefore, suppression of Aβ aggregation in blood vessels and its clearance are very important for the prevention of CAA. The mechanism by which Aβ is cleared remains unclear. However, some papers reported that small vessels of the brain play important roles in both the efflux across the blood-brain barrier [29] and the intramural perivascular drainage of Aβ in the interstitial fluid of the brain [30,31]. It was suggested that aging impairs perivascular drainage and increases Aβ accumulation and deposition along the basement membrane of small arteries [32]. The spread of Aβ from the basement membrane promotes the replacement of all tissue elements that make up the arterial wall with Aβ [33]. At a neuropathological level, in CAA, reduced smooth muscle cells are replaced with Aβ, the vessel wall becomes markedly thickened, and this causes reduced vessel compliance [9,[34], [35], [36], [37]]. It has been suggested that the loss of vessel compliance leads to the fragility of vessels and reduces perivascular clearance [38]. Additionally, as mentioned above, it is known that the pulsation of blood vessels is important for the perivascular drainage of interstitial fluid, and that decreasing vessel compliance reduces pulsation and impairs this drainage pathway [4,5].

When endothelial cells are damaged, they release pro-inflammatory cytokines, expanding neuroinflammation and secondary damage, also damaging the tight junctions of endothelial cells and the basal lamina, disrupting the blood-brain barrier and altering vessel physiology [[39], [40], [41]]. In our experiment, as the concentration of Aβ increased, the formation of actin dots over 10 μm^2^ increased, inducing endothelial cell death. This suggests that actin dot formation due to Aβ aggregation and accumulation may impair the cytoskeleton, impede the movement of endothelial cells, and cause their death.

In this study, we succeeded in real-time imaging of the Aβ aggregation process in the presence of vascular epithelial cells for the first time. The results revealed that Aβ aggregates firmly anchored the cells to the substrate and the cells were ruptured by their own migration force. The physical effects of Aβ aggregates on cells will provide a new insight for investigating the effects of Aβ on cells. In the future, it is expected that specific measures to prevent CAA will be found by elucidating the effect of Aβ aggregation on vascular endothelial cells from molecular and physical viewpoints.

## Declaration of competing interest

The authors declare no competing financial interests.

## Data Availability

No data was used for the research described in the article.
