# Supplementary material for: Amyloid β aggregation induces human brain microvascular endothelial cell death with abnormal actin organization
Source: Biochem Biophys Rep. 2021 Dec 15;29:101189. doi: 10.1016/j.bbrep.2021.101189 (PMC8685982; doi:10.1016/j.bbrep.2021.101189)
Supplement: Multimedia component 1 [file mmc1.pdf]

Supplementary Materials

Amyloid β aggregation induces human brain microvascular endothelial cell death with abnormal actin organization

Yushiro Take[a,b,c,1], Yusaku Chikai[a,1], Keiya Shimamori[a], Masahiro Kuragano[a], Hiroki Kurita[c], and Kiyotaka Tokuraku[a,*]

[a]Graduate School of Engineering, Muroran Institute of Technology, Hokkaido 050-8585, Japan

[b]Ohkawara Neurosurgical Hospital, Hokkaido 050-0082, Japan

[c]Saitama International Medical Center, Department of Cerebrovascular Surgery, Saitama 350-1298, Japan

1 These authors contributed equally.

*Address correspondence to: Tokuraku Kiyotaka, Ph.D. (tokuraku@mmm.muroran-it.ac.jp)

Supplementary Figures

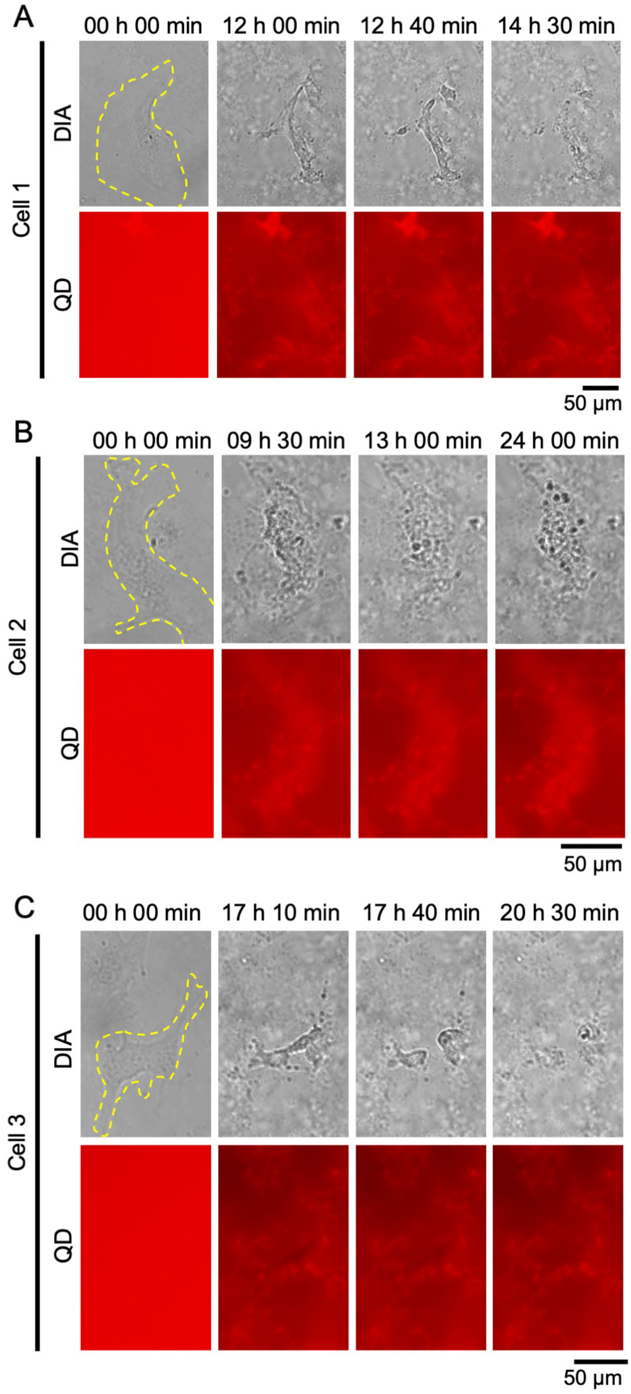

Sup. Fig. S1: Aβ aggregation and endothelial cell death under the same conditions as in Fig 1. hBMECs co-incubated with 20 μM $A\beta_{42}$ and 30 nM QDAβ were observed by an inverted fluorescence microscope. This figure shows another cell, not viewed in Fig. 1. The yellow dotted lines show the outline of endothelial cells.

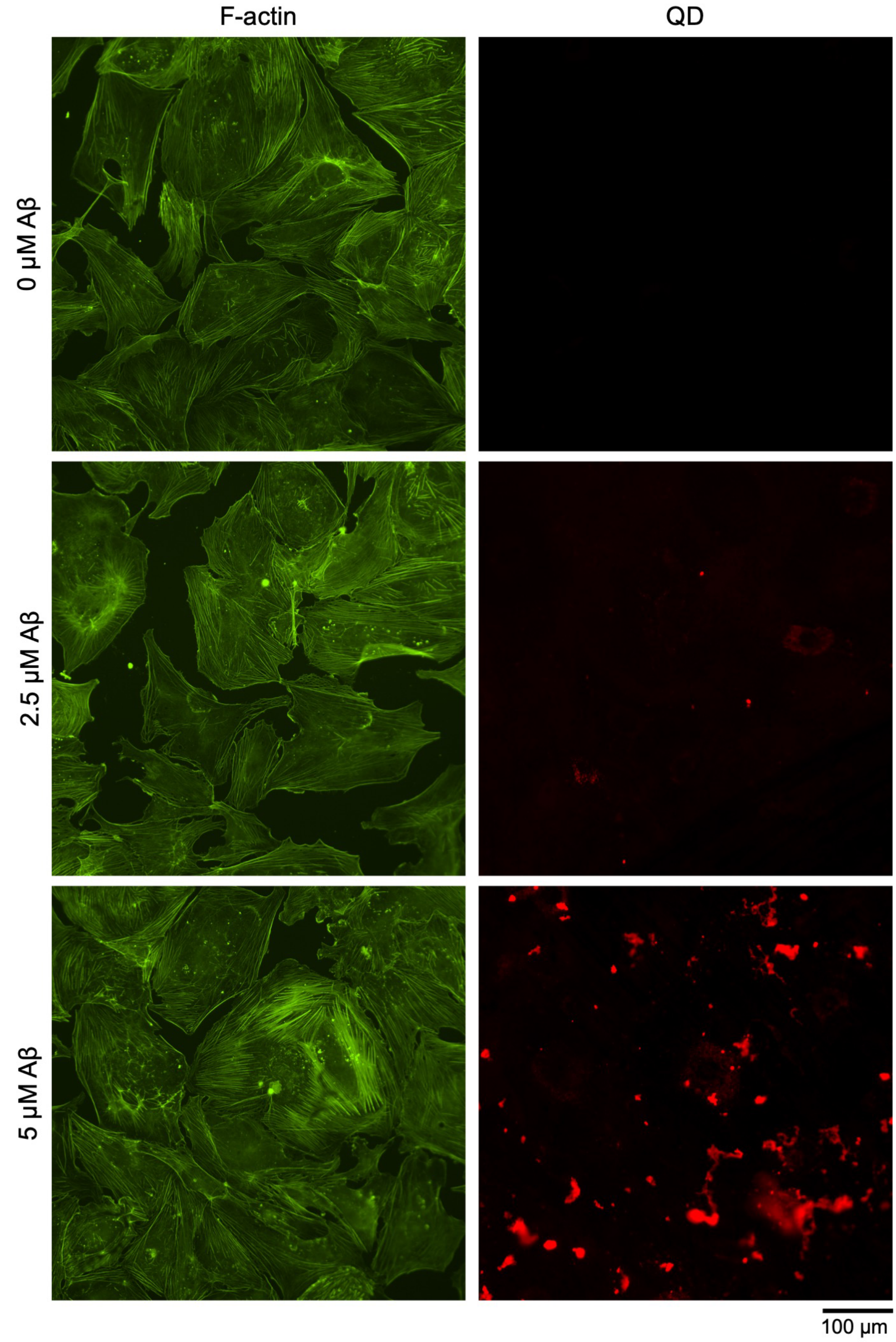

Sup. Fig. S2: Fluorescence images of F-actin in hBMECs and Aβ aggregates under the same conditions as in Fig 4. hBMECs were co-incubated with Aβ of each concentration and 30 nM QDAβ (red) for 24 h, then stained for F-actin (green) with Alexa 488 Phalloidin. Alexa 488 and QD were imaged by an inverted fluorescence microscope.

Supplementary Movie Legends

Sup. Movie S1. hBMECs were co-incubated with 20 μM $A\beta_{42}$ and 30 nM QDAβ. This movie shows that hBMECs induced cell death by Aβ aggregation. White arrowheads indicate hBMECs that caused cell death. Images were captured at a rate of 1 frame/10 min. Playback speed is 15 frames/sec. Still images are displayed in Fig. 1 and Fig. S1. Bar = 100 μm.

Sup. Movie S2. hBMECs were treated with 0.4% DMSO without $A\beta_{42}$ (control condition). Images were captured at a rate of 1 frame/10 min. Playback speed is 15 frames/sec. Bar = 50 μm.

Sup. Movie S3. hBMECs were co-incubated with 10 μM $A\beta_{42}$ and 30 nM QDAβ. This movie shows that hBMECs were anchored to the plate surface by Aβ aggregation. White arrowheads indicate points of cells anchored to the plate surface by Aβ aggregates. Images were captured at a rate of 1 frame/10 min. Playback speed is 15 frames/sec. Still images are displayed in Fig. 2A. Bar = 50 μm.

Sup. Movie S4. hBMECs were co-incubated with 5 μM $A\beta_{42}$ and 30 nM QDAβ. This movie shows that hBMECs were cut. White arrowhead shows a part of a cell where $A\beta_{42}$ aggregated and was broken off. Images were captured at a rate of 1 frame/10 min. Playback speed is 10 frames/sec. Still images are displayed in Fig. 2B. Bar = 50 μm.

Sup. Movie S5. hBMECs were co-incubated with 10 μM $A\beta_{42}$ and 30 nM QDAβ. This movie shows that Aβ aggregation induced holes in the cell monolayer. Images were captured at a rate of 1 frame/10 min. Playback speed is 15 frames/sec. Still images are displayed in Fig. 3. Bar = 100 μm.
